# Editorial: Cystic Fibrosis in Children

**DOI:** 10.3389/fped.2022.917221

**Published:** 2022-05-03

**Authors:** Bülent Taner Karadag, Elpis Hatziagorou, Alejandro Teper, Refika Ersu

**Affiliations:** ^1^Division of Pediatric Pulmonology, Faculty of Medicine, Marmara University, Istanbul, Turkey; ^2^3rd Pediatric Clinic, Aristotle University of Thessaloniki, Thessaloniki, Greece; ^3^Hospital General de Niños Ricardo Gutierrez, Buenos Aires, Argentina; ^4^Division of Respiratory Medicine, Children's Hospital of Eastern Ontario, University of Ottawa, Ottawa, ON, Canada

**Keywords:** cystic fibrosis, children, diagnosis, treatment, spirometry

Cystic fibrosis (CF) is the most common fatal genetic disease among Caucasians, occurring in 1 in 3,000 live births. According to the international registries, ~150,000 patients have a diagnosis of CF worldwide (1, 2). Based on the improvement in the nutritional and clinical care of the patients and setting CF centers with a multi-disciplined approach, general characteristics and the survival rates of CF patients have been changed remarkably. After the contribution of cystic fibrosis transmembrane conductance regulator protein (CFTR) modulator therapies, these changes will be more prominent in especially high-income countries (3). However, other factors in the diagnosis and treatment of patients in developing countries, even patients receiving CFTR modulators in high-income countries should be taken into account (4). Increased life expectancy, higher costs of new treatment modalities and a better understanding of genetics, call for a more sophisticated approach to these patients.

In contrary to a great amount of data on CF, many discrepancies still exist among countries and even between different centers. New treatment modalities have great potential to change the natural course of the disease, but due to the high economic cost of these treatments, inequality among patients in different socio-economic circumstances may increase. In a recent publication, only 12% of whole CF population in registries eligible for triple modulator therapies, were reported to have access to this modalities (5). An update of guidelines and research is needed for a standardized worldwide approach. For this purpose, we invited researchers to contribute to this collection focusing on main problems in the management of CF, especially emphasizing on the differences in approaches across the world.

This Research Topic gathers a collection 13 original and review articled providing crucial information on different aspects of CF. There is an interesting manuscript on the pathophysiology of CF, investigating the role of IL-22 and neutrophil proteins on the lung damage in the future (Renwick et al.). Early life concentrations of azurocidin and myeloperoxidase were found to have correlation with Brody scores at high resolution CT after 6 years of age. In addition to that, four other neutrophil associated proteins were negatively correlated with Brody scores. Also, first time in the literature, IL-22 levels in early age was correlated with increased lung damage. Identification of early signs of lung damage may help to improve the management of CF patients.

Early diagnosis of CF is still the mainstay of the management of the disease. In order to diagnose early and prevent complications, newborn screening programs have been implemented in many parts of the world. In this collection, different methods of newborn screening was reviewed (Coverstone and Ferkol). The threshold for defining a cut-off level of immunoreactive trypsinogen varies a lot between programmes. Adding genetic panels may additionally help to identify the patients with normal sweat test results. The advantage of screening in patients having mutations eligible for modulator therapies has also mentioned in this review. In animal models, primary prevention of CF was studied, which may lead to clinical trials in fetuses. Achieving primary prevention of CF has also been questioned in this article. Sweat testing is the main diagnostic tool for CF, evaluating the children after a positive newborn screening or patients with suggestive clinical findings. This procedure requires standardization and newer easy-to-perform techniques. These advances were discussed in another article in this collection (Gokdemir and Karadag). According to the recent guidelines, pilocarpine iontophoresis is the single accepted method, but accepted several others were also discussed in this review. Wearable sensors for ion exchange technology, skin wipe test for capillary electrophoresis are mentioned as newer methods.

Another series of investigations in this Research Topic, include reports on exercise techniques and different methods of measuring lung function. Oscillometry and multiple breath-washout techniques including lung clearence index (LCI) may give additional information about the lung health in CF. Although the role of LCI in clinical trials has been well-established, its role in daily care is not well-known. In an article from this collection, the authors reviewed the usage of LCI and cardiopulmonary exercise testing in daily care (Hatziagorou et al.). Although spirometry is the well-known method to follow patients, especially in early-stage disease newer methods may be more helpful. LCI is more sensitive to detect impairment of peripheral airways and prevent future damages than spirometry. Also LCI measurements shortly after birth is correlated with respiratory rate later in life and, also predicts the first Pseudomonas colonization. In addition to being expensive and time-consuming especially for severe patients, for LCI there is still a need for validity, age-specific reference values and more data for LCI in clinical follow up of the patients. Exercise and increased muscle strength are also important features for improving the management of CF. Handgrip strength was shown to have an association with lung function (Adair et al.). In this research, exercise program at home as a quality improvement project was shown to result in significant improvement in hand grip strength. In another research in this collection, the authors suggested LCI as a better tool than IOS for patients with CF (Postek et al.).

Early detection of CF-related diabetes (CFRD) is so important, continuous glucose monitoring seems to more useful than oral glucose tolerance test (OGTT) in an article from this collection and the authors suggest continuous monitoring should be included in the guidelines in the near future (Gojsina et al.). There are still limited data on allergic bronchopulmonary aspergillosis in CF. Diagnosis can be difficult in some cases. In addition to these challenges, current and future treatment options like monoclonal antibodies are widely discussed in a review (Sunman et al.).

Other breakthroughs introduced in this collection focus on new treatment modalities including triple therapy and the real life experience that will give insights about the future of the management. In a real-life study among patients above 12 years of age, long-term lumacaftor/ivacaftor treatment improved lung function, nutritional status, and sweat chloride levels especially in younger patients (Bui et al.). In another article, short-term effects of triple combination on glucose tolerance has been reported (Korten et al.). The effect of modulator therapies is still not clear. In this observational study, glucose levels of OGTT was found to be improved and the authors suggest this therapy might prevent CFRD. Also, nasal epithelial cell models seem to be useful in functional profiling of CFTR-directed therapies and enables a better understanding for personalized medicine (Park et al.). Culturing cells from nasal brushings instead of rectal or nasal biopsies also gives a non-invasive nethod for sampling.

During pandemic, appointments were tried to be converted to telemedicine visits and the need for digital health and using telemedicine in the management of CF became so clear (Hendra et al.). In this survey, more than 80% of the patients were satisfied with telemedicine visits but disparities in language and access to internet seems to cause additional problems.

In conclusion, this collection covers different aspects of current approach to CF and adds valuable information to the literature. Great amount of work published from all parts of the world will result in a better understanding of the changing face of CF and hopefully decrease the inequity among countries.

## Author Contributions

BK prepared the manuscript. EH, AT, and RE reviewed the manuscript. All authors contributed to the article and approved the submitted version.

## Conflict of Interest

The authors declare that the research was conducted in the absence of any commercial or financial relationships that could be construed as a potential conflict of interest.

## Publisher's Note

All claims expressed in this article are solely those of the authors and do not necessarily represent those of their affiliated organizations, or those of the publisher, the editors and the reviewers. Any product that may be evaluated in this article, or claim that may be made by its manufacturer, is not guaranteed or endorsed by the publisher.

## References

[1] Orenti A Zolin A Jung A van Rens J. ECFSPR Annual Report 2019. Karup: European Cystic Fibrosis Society (2021).

[2] Cystic Fibrosis Foundation. Cystic Fibrosis Foundation Patient Registry 2020 Annual Data Report. Bethesda, MD: Cystic Fibrosis Foundation (2021).

[3] Lopes-Pacheco M. CFTR modulators: the changing face of cystic fibrosis in the era of precision medicine. Front Pharmacol. (2020) 10:1662. 10.3389/fphar.2019.01662 32153386 PMC7046560

[4] Zampoli M Kashirskaya N Karadag B da Silva LV Paul GR Noke C. Global access to affordable CFTR modulator drugs: time for action! *J Cyst Fibros*. (2022). 10.1016/j.jcf.2022.03.006. [Epub ahead of print]. 35341695

[5] Guo J Garratt A Hill A. Worldwide rates of diagnosis and effective treatment for cystic fibrosis. J Cyst Fibros. (in press). 10.1016/j.jcf.2022.01.009 35125294

